# Functional Characterization of Colon Cancer-Associated Mutations in *ADAM17*: Modifications in the Pro-Domain Interfere with Trafficking and Maturation

**DOI:** 10.3390/ijms20092198

**Published:** 2019-05-04

**Authors:** Egor Pavlenko, Anne-Sophie Cabron, Philipp Arnold, Jan Philipp Dobert, Stefan Rose-John, Friederike Zunke

**Affiliations:** 1 Institute of Biochemistry, Christian-Albrechts-Universität zu Kiel, 24118 Kiel, Germany; stu119549@mail.uni-kiel.de (E.P.); acabron@biochem.uni-kiel.de (A.-S.C.); stu118262@mail.uni-kiel.de (J.P.D.); rosejohn@biochem.uni-kiel.de (S.R.-J.); 2 Institute of Anatomy, Christian-Albrechts-Universität zu Kiel, 24118 Kiel, Germany; p.arnold@anat.uni-kiel.de

**Keywords:** ADAM17, colon cancer, metalloprotease, shedding, tumor necrosis factor, EGF-receptor, secretory pathway, trafficking of membrane proteins

## Abstract

Colorectal cancer is one of the most commonly diagnosed malignancies in the Western world and is associated with elevated expression and activity of epidermal growth factor receptors (EGF-R). The metalloproteinase ADAM17 is involved in EGF-R activation by processing EGF-R ligands from membrane-bound pro-ligands. Underlining the link between colon cancer and ADAM17, genetic intestinal cancer models in ADAM17-deficient mice show a reduced tumor burden. In this study, we characterize point mutations within the *ADAM17* gene found in the tissue of colon cancer patients. In order to shed light on the role of ADAM17 in cancer development, as well as into the mechanisms that regulate maturation and cellular trafficking of ADAM17, we here perform overexpression studies of four ADAM17 variants located in the pro-, membrane-proximal- and cytoplasmic-domain of the ADAM17 protein in ADAM10/17-deficient HEK cells. Interestingly, we found a cancer-associated point mutation within the pro-domain of ADAM17 (R177C) to be most impaired in its proteolytic activity and trafficking to the cell membrane. By comparing this variant to an ADAM17 construct lacking the entire pro-domain, we discovered similar functional limitations and propose a crucial role of the pro-domain for ADAM17 maturation, cellular trafficking and thus proteolytic activity.

## 1. Introduction

Colorectal cancer (CRC) is one of the most common cancer types with a total of ~137,000 new cancer cases and ~50,000 deaths each year in the United States [1]. Whereas only 20% of the cases are familiar [2], risk factors include chronic intestinal inflammation [3] as well as overexpression and increased activity of the epidermal growth factor receptors (EGF-R) [4]. EGF-R, which is also known as ErbB1, belongs to a superfamily of transmembrane tyrosine kinase receptors further including ErbB2, ErbB3 and ErbB4. Known ligands to induce EGF-R activity include the epidermal growth factor (EGF), heparin-binding EGF (Hb-EGF), the transforming growth factor-α (TGF-α), amphiregulin (AREG), epiregulin (EREG) and β-cellulin (BTC) [4]. EGF-R signaling regulates differentiation, proliferation, gastric barrier function and cellular survival, which highlights its important role in health, but also disease, under which its dysfunction has been shown to be involved in various epithelial cancers types [4,5]. The protein ADAM17 belongs to the family of a disintegrin and metalloprotease and was first described as a tumor necrosis factor (TNF) α-converting enzyme [6]. So far over 80 substrates are described for the metalloprotease, including the interleukin-6 receptor (IL-6R), TNFα, both TNF receptors, as well as most of the EGF-R ligands, which are synthesized as a membrane-bound precursor: TGF-α, AREG, Hb-EGF, EREG [6,7,8,9]. Due to this variety of substrates, ADAM17 is involved in various physiological and pathophysiological processes like immunity, regeneration, development, inflammation or tumorigenesis [6,7,10,11,12]. Moreover, the involvement of ADAM17 in colorectal cancer has been shown in a hypomorphic ADAM17 mouse model, which exhibits significantly reduced ADAM17 protein and no detectable ADAM17 cleaving activity [10]. In a recent study, it was shown that the absence of ADAM17 in a genetic intestinal cancer background (APC^Min/+^) resulted in less intestinal tumors, which were also of low-grade dysplasia [13]. While it was demonstrated that colorectal tumor formation requires cleavage of IL-6R by ADAM17 to mediate soluble (s)IL-6R-dependent IL-6 trans-signaling [13,14], it further underlines the significance of ADAM17 in CRC pathology. Importantly, also other molecular actors, as for example the serine hydrolase monoacylglycerol lipase (MAGL) [15] or transcription factor SATB2 [16] have been recently identified and described as potential therapeutic targets in CRC oncogenesis. Hence, pharmacological inhibition of MAGL was able to reduce tumor volume, which was further associated with downregulation of fibroblast growth factor-2 (FGF-2) and vascular endothelial growth factor (VEGF) [15]. Interestingly, VEGF expression can be regulated by IL-6 trans-signaling [17] and cleavage of the VEGF-receptor (VEGF-R2) has also been shown to be mediated by ADAM17 [9]. This moreover illustrates the complexity of CRC and the central role of the metalloprotease ADAM17 in intestinal cancer development.

Although ADAM17 has been shown to be involved in various important cell functions as well as in cancer metabolism, its maturation and regulation of proteolytic function have only been poorly understood. To date there is no protein structure of the full ADAM17 protein available, but the protease, which is a type I transmembrane protein, can be subdivided in structurally and functionally different protein domains: a signal peptide (SP) followed by a pro-domain (PD), a catalytic domain (CATALYTIC), a disintegrin domain (DD), a membrane-proximal domain (MPD), a transmembrane domain (TM) and a cytoplasmic domain (CD) [6,7]. ADAM17 is expressed as an inactive proenzyme and needs to be post-translationally modified in order to be active. The pro-domain has been shown to be an important regulator of proteolytic function since it has been proposed to act as a chaperone [18] until it is removed by furin-like proprotein convertases in the Golgi apparatus. Hence, two furin cleavage sites within ADAM17 have been proposed: one located between the pro- and the catalytic-domain (downstream (ds)) and the second one within the C-terminal part of the pro-domain (upstream (us)) [19]. However, due to its high affinity, the pro-domain can stay associated with the catalytic domain even after furin cleavage [20], keeping the protease in an inactive state until it is released from the main protein [20]. Hence, a recombinant pro-domain has been shown to be a highly specific and potent inhibitor of ADAM17 activity in vitro and in vivo [20,21]. The MPD has been proposed to be important for substrate recognition in particular of IL-6R [22]. In contrast, the impact of the cytoplasmic-domain on protease function is still under debate, since CD-deletion mutants have shown controversial shedding activities in different studies [23,24,25,26,27,28], which might be explained by the use of various cell lines and expression constructs [28]. Nonetheless, the physiological importance of the CD has been implied, since posttranslational phosphorylation can occur within this protein domain. Thus, modified ADAM17 function can be observed after treatment of cells with phorbol ester (PMA) [29]. Under physiological conditions, for instance, MAPKs and polo-like kinase 2 (PLK2) lead to an increase in ADAM17 phosphorylation [30,31,32,33]. As such, a phosphorylation at threonine 735 has shown to rapidly activate ADAM17 activity [30].

In the present study, we screened databases containing somatic *ADAM17* mutations in tumor samples from colon cancer patients revealing coding mutations within all domains of ADAM17. We here characterize four naturally occurring point mutations within three different protein domains to learn about the importance and impact of each individual mutation in the respective domains. Since it was already shown that a mutation in the catalytic domain leads to proteolytic inactive ADAM17 [34], we focus on point mutations in the pro-, membrane-proximal- as well as cytoplasmic domain and analyze maturation, trafficking to the cell membrane and proteolytic activity of the ADAM17 variants.

Our data provide evidence that overall colon cancer-associated ADAM17 variants in the MPD and CD lack sufficient cell surface trafficking, but vary only slightly in their proteolytic activity in comparison to the wildtype (wt) construct. Only a mutation found in the pro-domain of ADAM17 majorly affects protein maturation, localization to the cell membrane and proteolytic activity. Since this point mutation behaved similar to an ADAM17 variant lacking the entire pro-domain, we propose a crucial function of this ~200 amino acid long pro-peptide for proper ADAM17 maturation and function in health and disease as discussed here for cancer development.

## 2. Results

### 2.1. Mutations in ADAM17 Are Associated with Colon Cancer

Databases (IntOGen, COSMIC, TCGA and ICGC) listing somatic mutations in cancer tissue were searched for mutations within the *ADAM17* gene in colon cancer samples. The COSMIC database shows a total analysis of 48,028 unique samples of various cancer tissues that were analyzed for mutations within *ADAM17*. Overall, 189 unique samples were found with coding ADAM17 mutations: https://cancer.sanger.ac.uk/cosmic/gene/analysis?ln=ADAM17.

Breaking these numbers down to individual tissues, 2331 cancer samples taken from the large intestine, which includes the caecum and colon, resulted in 40 positive samples harboring a coding mutation within *ADAM17* (incidence: 1.7%). Comparing all analyzed tissues, only within the small intestine a higher incidence of *ADAM17* mutations was found, but the samples size was much smaller (incidence: 1.9%, 52 samples, 1 positive). Thereby, mutations found within the large intestine constitute 55% of missense, 17.5% of synonymous, 10% of nonsense substitutions and 7.5% of frameshift deletions.

**Figure 1:**
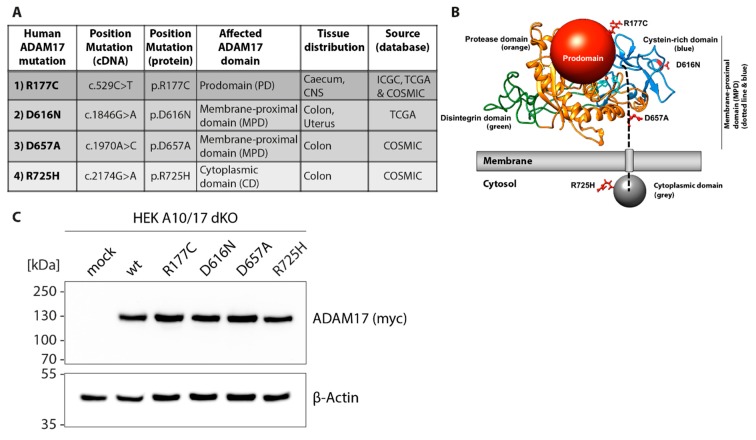
Overview and expression of human ADAM17 variants associated with colon cancer within the pro-, membrane-proximal and cytoplasmic domain. (**A**) Overview of colon cancer-associated ADAM17 variants analyzed in this study, showing somatic point-mutations found in *ADAM17* in genome screenings of tumor tissue as listed in the ICGC, TCGA and COSMIC databases. Positions of mutations in cDNA and amino acid sequence, as well as their domain localization inside the ADAM17 protein, are indicated in the table. (**B**) Structural model of ADAM17 showing the protein domains in different colors and point mutations in red: protease/catalytic domain (orange); pro-domain (red ball) with highlighted R177C mutation; disintegrin domain (green); membrane-proximal domain consisting of stalk region (dotted black line) and cysteine-rich domain (blue) with D657A and D616N mutations; cytoplasmic domain (grey ball) with R725H. ADAM17 was modeled using ADAM10 (pdb: 6BE6) as a template in Swiss-Model Expasy. (**C**) Cloning of respective colon cancer-associated ADAM17 variants in pcDNA3.1 expression vector and overexpression in ADAM10/ADAM17 double-deficient HEK cells (HEK A10/A17 dKO). Representative immunoblot of overexpressed ADAM17 wildtype (wt) and colon cancer-associated variants exhibiting equal expression level. All ADAM17 constructs carry C-terminal myc-tag, which was used for detection (anti-myc). β-Actin was used as a loading control.

In this study, we focused on four missense mutations found in the pro-, membrane-proximal- and cytoplasmic-domain (Figure 1A). The first ADAM17 mutation we here analyze is found in the pro-domain of the protein (c.529C > T; p.R177C). At the protein level, a positively charged arginine is exchanged by a cysteine. Interestingly, this mutation was not only found in cancer tissue of the caecum, but also in another patient within a glioma (astrocytoma grade IV; COSMIC database, Sanger Institute) (Figure 1A). In total, we analyzed two mutations found in the membrane-proximal domain: p.D616N (c.1846G > A) and p.D657A (c.1979A > C). After the introduction of the point mutation, both ADAM17 variants lose the negative charge of the aspartic acid. As indicated in the ADAM17 protein model (Figure 1B), the membrane-proximal domain consists of a stalk region (dotted black line), where the D657A point mutation can be found and a cysteine-rich domain (blue), in which the D616N mutation is located. We further analyzed one cancer-associated mutation found in the cytoplasmic domain (dark grey): R725H (c.2174G > A) (Figure 1A,B). Since there is no crystal structure of the entire ADAM17 protein available, we produced a homology model on the basis of the crystal structure of the closely related ADAM10 (pdb: 6BE6) and depicted the different domains (Figure 1B). Protein domains lacking structural information (pro-domain, stalk region, cytoplasmic domain) are shown as spheres or dotted line (Figure 1B).

The ADAM17 variants analyzed in this study were generated by mutagenesis PCR and introduced into a wildtype mADAM17 pcDNA3.1 plasmid. Moreover, the constructs were C-terminally tagged with a myc-sequence. We reconstituted the respective ADAM17 variants and wildtype into ADAM10 and ADAM17 double knock-out HEK cells (HEK dKO) by transient overexpression (Figure 1C). Equal expression levels of the ADAM17 variants was verified by SDS-PAGE and Western blot using an anti-myc antibody (Figure 1C).

### 2.2. Colon Cancer-Associated ADAM17 Variants Differ in Their Proteolytic Activity

**Figure 2:**
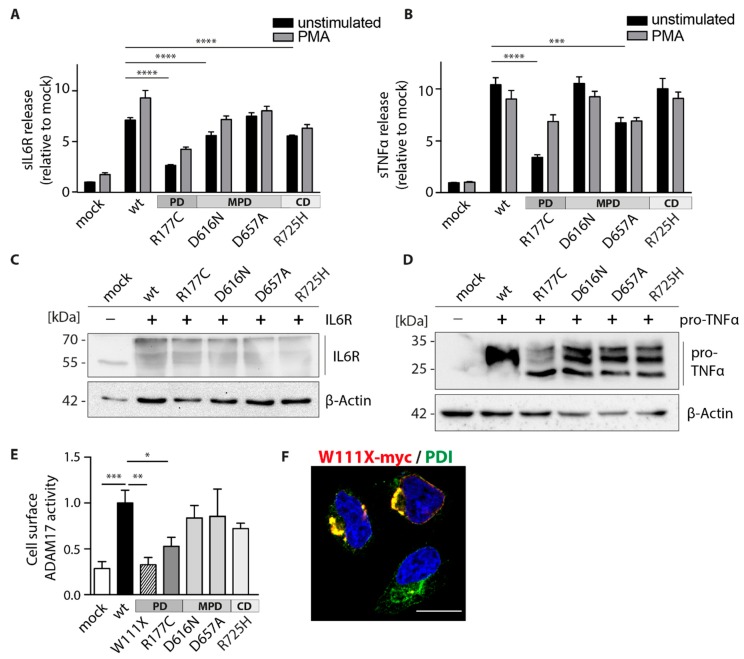
Proteolytic activity of colon cancer-associated ADAM17 variants. Enzyme-Linked Immunosorbent Assay (ELISA) measuring soluble (s)IL-6R (**A**) and TNFα (**B**) in supernatants of dKO HEK cells expressing wt and colon cancer-associated ADAM17 variants. Values were normalized to mock-transfected cells. Statistical significances are shown in comparison to wt utilizing a one-way ANOVA together with a Dunnett’s multiple comparison test. *** *p* < 0.005, **** *p* < 0.001. *n* = 4, from four independent transfection rounds with three technical replicates. Representative immunoblots of equal IL-6R (**C**) and pro-TNFα (**D**) expression after co-transfection with ADAM17 variants. β-Actin was used as a loading control. (**E**) Cell surface proteolytic activity of ADAM17 variants in living HEK dKO cells using a fluorogenic TNFα peptide. Increase of fluorescent signal as a result of ADAM17 cleavage was detected every 30 s over 120 min. Activity was calculated as the area under the curve and normalized to wt. Colon cancer-associated ADAM17 W111X mutation is truncated after amino acid 111, lacking the catalytic domain and was used as a negative control. A one-way ANOVA together with a Dunnett’s multiple comparison test was applied. * *p* < 0.05, ** *p* < 0.01, *** *p* < 0.005. *n* = 4–7 derived from 4–7 independent transfection rounds. (**F**) Immunofluorescence of dKO HEK cells overexpressing ADAM17 W111X-myc, shown in red (anti-myc) and protein disulfide isomerase (PDI) as an ER marker in green. Scale bar: 10 µm.

To analyze the consequences of the respective ADAM17 colon cancer-associated point mutations on their proteolytic activity, functional analyses were performed in the following. Hence, HEK dKO cells were co-transfected with the respective ADAM17 variants as well as the ADAM17 substrates: IL-6 receptor (IL-6R) and pro-TNFα. The shed (soluble) substrates (sIL-6R and sTNFα) found in the supernatants of cells were measured by ELISA assay (Figure 2A,B). HEK dKO cells reconstituted with ADAM17 wt showed significantly higher shedding activity in comparison to the mock-transfected control indicated by increased sIL-6R and sTNFα level (Figure 2A,B). To further stimulate ADAM17-depended shedding we incubated the cells with PMA (200 nM, 2 h), an activator of the protein kinase C, which has also shown to activate ADAM17 [29]. The equal expression of the substrates was always verified by SDS-PAGE and Western blotting (Figure 2C,D). Most strikingly, the shedding activity of the R177C mutation located within the pro-domain of ADAM17 is highly diminished for both substrates (IL-6R and pro-TNFα) (Figure 2A,B). Interestingly, all other mutants within the membrane-proximal- and cytoplasmic-domains, show the general ability to still shed both substrates, although in some cases with lesser efficiency. Hence, the D616N and R725H mutants revealed significantly lower levels of sIL-6R (Figure 2A) compared to the wildtype, but exhibited normal shedding activity towards pro-TNFα (Figure 2B). In contrast, the D657A mutation displayed normal shedding activity towards IL-6R (Figure 2A), but diminished cleavage of pro-TNFα (Figure 2B).

To further characterize the impact of the individual *ADAM17* mutations on the protein’s function and cellular localization, we performed a cell surface activity assay using a fluorogenic TNFα peptide incubated on live HEK dKO cells (Figure 2E). Thus, the fluorescent signal is only detectable if catalytically active ADAM17 is present at the cell surface. In comparison to the wildtype, cancer-associated mutations of ADAM17 within the membrane-proximal- (D616N and D657A) and the cytoplasmic-domain (R725H) showed a slightly reduced, but not significantly impaired cell surface activity (Figure 2E). Interestingly, the mutant within the pro-domain (R177C) exhibited diminished cell surface activity (Figure 2E). As an additional negative control next to the mock-transfected cells, we analyzed another large intestine cancer-associated mutant (p.W111X, c.332G > A, COSMIC, Sanger Institute). This variant is truncated after amino acid 111, consisting only of the signal peptide and partly of the pro-domain and therefore shows no ADAM17 activity (Figure 2E). Immunofluorescence staining of the W111X mutant together with an ER marker (PDI) revealed enlarged vesicles and indicating inefficient trafficking of this mutant out of the ER to the cellular compartment, such as the cell surface (Figure 2F).

Overall, these results suggest that mutations within the membrane-proximal domain may alter recognition of the substrates and therefore lead to variable shedding activity towards IL-6R and pro-TNFα. However, the mutation located in the ADAM17 pro-domain (R177C) affected the function of ADAM17 in the most severe manner, indicated by a reduced substrate shedding as well as diminished cell surface cleavage activity.

### 2.3. Cellular Localization Is Altered in Colon Cancer-Associated ADAM17 Variants

**Figure 3:**
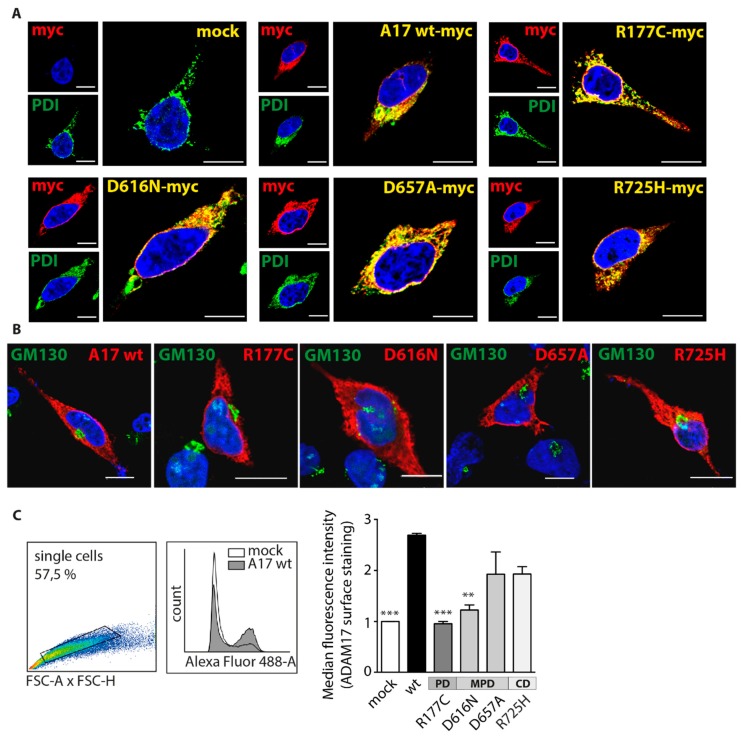
Impact of colon cancer-associated ADAM17 variants on cellular localization. (**A**,**B**) Representative immunofluorescence pictures of ADAM17 variants overexpressed in HEK dKO cells (anti-myc, red) co-stained for the ER (**A**; PDI, green) and the Golgi (**B**; GM130, green). Scale bar: 10 µm. (**C**) Cell surface staining of ADAM17 variants by FACS analysis. An extracellular (N-terminal) antibody (10.1) was used for ADAM17 staining. Single cells were gated and plotted for secondary antibody signal (Alexa Fluor 488). Median fluorescence intensity is shown normalized to mock-transfected cells. Statistical significance was determined by using a one-way ANOVA together with a Dunnett’s multiple comparison test and is shown in comparison to wt. ** *p* < 0.01, *** *p* < 0.005. *n* = 3 derived from three independent transfection rounds and FACS analyses.

Since cell surface localization is crucial for ADAM17 shedding activity, we next analyzed cellular localization of the respective ADAM17 variants. Following, we performed immunofluorescence studies of transfected cells (Figure 3A,B), as well as cell surface flow cytometry analyses (Figure 3C). HEK dKO cells reconstituted with the respective ADAM17 variants were stained for the protease by utilizing a myc (red) antibody and co-stained for the ER-marker PDI (green) (Figure 3A) or Golgi-residual protein GM130 (green) (Figure 3B). Co-localization of ADAM17 signal and PDI is indicated in yellow and implies localization of all ADAM17 variants mainly in the ER (Figure 3A). Moreover, there seems to be no interference of any ADAM17 construct with the Golgi-apparatus (Figure 3B). As a cell surface protein, ADAM17 is matured within the secretory pathway and will thereby always be found in intracellular compartments. Surprisingly, it was difficult to detect ADAM17 wt protein on the cell surface utilizing confocal imaging (Figure 3A,B), which might be due to insufficient sensitivity of this method. We, therefore, performed a more sensitive and quantitative analysis to detect ADAM17 on the cell surface utilizing an ADAM17 antibody binding to the extracellular *N*-terminal part of the protein and performed FACS analyses (Figure 3C). After gating single cells (Figure 3C, left panel), the median fluorescent intensity (MFI) was determined and normalized to mock-transfected cells. None of the ADAM17 variants reached the cell surface in the same amount as the wildtype (Figure 3C). Strikingly, the pro-domain mutant (R177C), as well as the MPD-variant D616N, is significantly decreased in cell surface localization, indicating a trafficking defect of both ADAM17 variants (Figure 3C).

Taken together, our data indicate that colon cancer-associated ADAM17 mutations negatively affect protein maturation and transport via the secretory pathway. Especially mutations in the pro-domain, as well as in the early (N-terminal) MPD seem to affect trafficking to the cell surface most severely. This trafficking defect is most likely the reason for impaired ADAM17 shedding activity, which is mediated on the cell surface.

### 2.4. A Colon Cancer-Associated ADAM17 Mutation within the Pro-Domain Is Catalytically as Inactive as an ADAM17 Variant Lacking the Entire Pro-Domain

**Figure 4:**
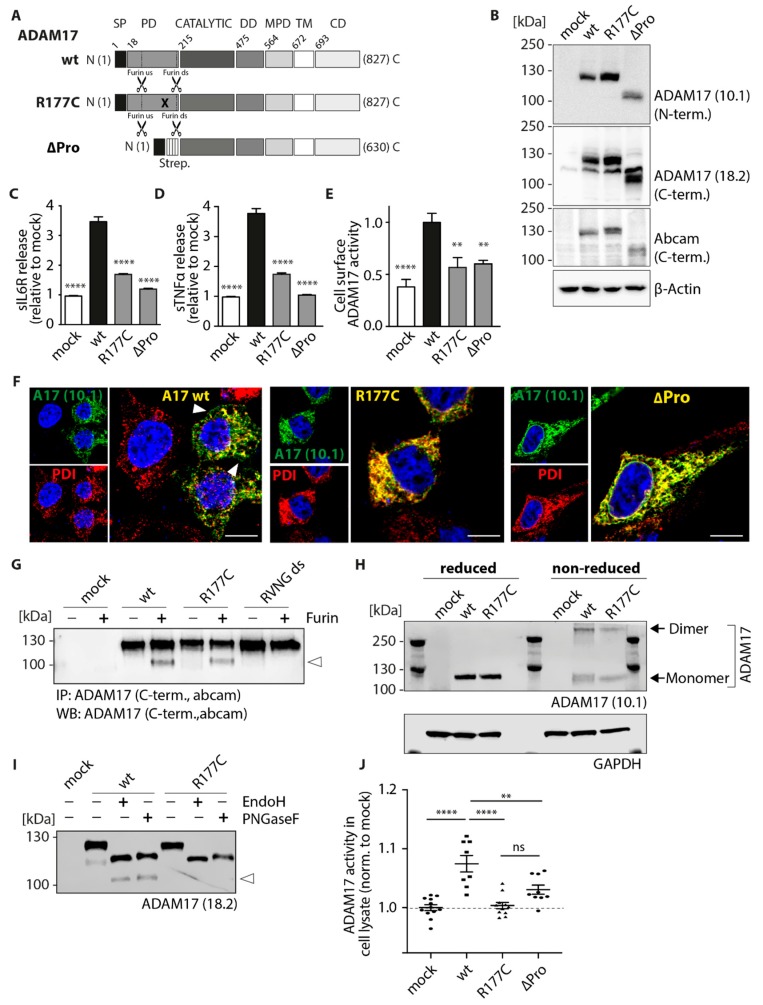
The ADAM17 pro-domain: function and impact of mutations on protease activity and cellular localization. (**A**) Schematic overview of linear ADAM17 protein and its domains. SP, signal peptide; PD, pro-domain; CATALYTIC, catalytic domain; DD, disintegrin domain; MPD, membrane-proximal domain; TM, transmembrane domain; CD, cytoplasmic domain. Shown is the R177C mutation which is localized in the pro-domain (indicated by a black cross) and the ΔPro variant, lacking the entire pro-domain. The ΔPro variant carries a triple streptavidin (strep)-tag between the signal peptide and the catalytic domain. Furin cleavage sites (us, upstream and ds, downstream) are indicated by scissors. (**B**) Immunoblots of ADAM17 variants showing equal expression of all constructs using N-terminal and C-terminal antibodies. β-Actin was used as a loading control. IL-6R (**C**) and pro-TNFα (**D**) shedding ELISA assay using supernatants of HEK dKO cells co-transfected with ADAM17 variants and substrates (IL-6R or pro-TNFα). The results were normalized to the mock-transfected control. Statistical significance is shown in comparison to ADAM17 wt. *n* = 3 derived from three independent transfection rounds measured in triplicates. (**E**) Shedding activity of ADAM17 variants on the cell surface of live HEK dKO cells towards fluorogenic TNFα peptide. Fluorescence was measured every 30 s over a total time of 120 min. The results shown are the area under curve normalized to the wt value. Statistical significance is shown in comparison to ADAM17 wt. *n* = 3 derived from three independent transfection rounds measured in triplicates. (**F**) Immunofluorescence staining of ADAM17 wt, R177C and ΔPro variants in HEK dKO cells. ADAM17 was detected by utilizing an N-terminal antibody (10.1, green) and the ER was co-stained using an anti-PDI antibody (red). White arrow indicates cell surface localization of ADAM17 wt protein. Scale bar: 10 µm. (**G**) Representative immunoblot of ADAM17 after performing a furin cleavage assay. Lysates of transfected HEK dKO cells were immunoprecipitated with a C-terminal anti-ADAM17 antibody from abcam and afterwards incubated with 1 U recombinant furin. After furin-cleavage of the pro-domain, a second band appears at ~100 kDa, corresponding to the mature form of ADAM17 (indicated by a white arrow). A furin-resistant ADAM17 mutant (RVNG ds) was used as an assay control. (**H**) Immunoblot of ADAM17 wt and R177C under reducing and non-reducing conditions. ADAM17 was detected using anti-ADAM17 (10.1) antibody. (**I**) Immunoblot of ADAM17 after protein deglycosylation assay. HEK dKO cell lysates overexpressing ADAM17 wt or R177C variant were treated with EndoH or PNGaseF enzyme for 1 h at 37 °C. ADAM17 was detected using the 18.2 ADAM17 antibody. After deglycosylation the ADAM17 band shifts from ~130 kDa to ~120 kDa. An additional band appearing only in the ADAM17 wt sample corresponds to the mature form of ADAM17 (~100 kDa; indicated by a white arrow). (**J**) ADAM17 activity assay in whole cell lysate of HEK dKO transfected with ADAM17 wt, R177C and ΔPro variants by fluorogenic TNFα peptide cleavage assay. The activity of whole cell ADAM17 activity was normalized to mock control. *n* = 3. All statistical analyses (C,D,E,J) were performed by using a one-way ANOVA together with a Dunnett’s multiple comparison test. ** *p* < 0.01, **** *p* < 0.001.

Interestingly, our data indicate major functional impairments of the cancer-associated mutant R177C located within the pro-domain. Since the pro-domain of ADAM17 has been shown to exhibit chaperone function [18], we wanted to study the R177C variant in more detail and compare the single point-mutation with an ADAM17 construct lacking the entire pro-domain (ΔPro) (Figure 4A). Hence, we next performed expression, glycosylation, maturation, cellular localization and functional analyses (Figure 4). Expression of ADAM17 wt, R177C and ΔPro variant, which moreover contains a triple streptavidin (strep)-tag between the signal peptide and the catalytic domain, in HEK dKO cells was verified by SDS-PAGE and Western blot using an N-terminal and two C-terminal anti-ADAM17 antibodies (Figure 4B). All ADAM17 variants showed equal expression level (Figure 4B). In comparison to the ADAM17 wt construct, the ΔPro variant is detected at ~100 kDa, which corresponds to the size of the mature ADAM17 protein lacking the pro-peptide. We further analyzed the shedding activity of the R177C mutant in comparison to the ΔPro variant and could detect diminished proteolytic activity of both ADAM17 variants towards IL-6R (Figure 4C) and proTNFα (Figure 4D). Moreover, we analyzed cell surface ADAM17 activity using the fluorogenic peptide-based activity assay: the R177C, as well as the ΔPro mutant, exhibited significant lower cell surface ADAM17 activity compared to the wildtype (Figure 4E). To analyze cellular localization of these two ADAM17 variants, we co-stained ADAM17 using a C-terminal antibody (10.1; green) and an ER marker (PDI; red) (Figure 4F). By utilizing this C-terminal antibody instead of the myc-antibody (Figure 3A), we were able to detect the localization of the ADAM17 wt to the cell membrane (Figure 4F, left panel, white arrows). In comparison to the wt, the R177C as well as the ΔPro mutant show high colocalization with the ER-marker PDI, indicating enhanced ER-localization and thus impaired transport to the cell surface (Figure 4F, middle and right panel). Previous studies have shown that ADAM17 needs to be processed by furin, which is cleaving the pro-domain at two sites in order for it to be removed, so the enzyme can deploy its full proteolytic activity [19]. Hence, we wanted to analyze if the R177C mutation interferes with the furin cleavage. Therefore, immunoprecipitated ADAM17 was incubated with recombinant furin and analyzed by immunoblot (Figure 4G). As a negative control, the furin-resistant mutant RVNG ds was used, which contains a mutation within the downstream furin cleavage motif (RVKR to RVNG). Thereby this mutant is no longer able to be processed by furin (Figure 4G). The immunoblot stained by a C-terminal ADAM17 antibody showed a predominant band at ~120 kDa, which refers to the non-mature form of ADAM17 and a second band for the wt and R177C mutant at ~100 kDa after incubation with furin (Figure 4G). This band most likely corresponds to the mature form of ADAM17 (indicated by an arrow) and suggests that the R177C mutation does not interfere with furin cleavage in an in vitro approach. Since the insertion of a cysteine residue can introduce novel disulfide bridges within the ADAM17 molecules or even between different ADAM17 proteins potentially leading to dimerization of the protein, we analyzed the effect of the cysteine insertion under non-reducing conditions. Therefore, overexpressed ADAM17 variants were analyzed by SDS-PAGE and immunoblotting under reducing and non-reducing conditions (Figure 4H). Interestingly, we could detect a band for the ADAM17 monomer (~130 kDa) and ADAM17 dimer (~260 kDa), but no differences between the wt and the R177C variant. Since three potential *N*-glycosylation sites were predicted within the pro-domain of ADAM17 [35], we further characterized the glycosylation pattern of the R177C variant in comparison to the ADAM17 wt (Figure 4I). Although the sensitivity towards endoglycosidase H (EndoH) and peptide:*N*-glycosidase F (PNGaseF) was comparable between wt and R177C, the ADAM17 wt sample exhibited an additional protein band at around ~100 kDa, which most likely corresponds to the mature form of ADAM17 (lacking the pro-domain; indicated by an arrow) (Figure 4I). The absence of this smaller protein band of the R177C mutant indicates a lack of maturation and processing of the protease under physiological conditions within the cell. This observation raises the question of whether the R177C mutant is constitutively inactive or simply impaired in cell surface trafficking leading to diminished ADAM17 activity. To further investigate this, we conducted a fluorogenic peptide cleavage assay on whole cell lysate of transfected HEK dKO cells (Figure 4J). Interestingly, only ADAM17 wt-substituted cell lysates exhibited ADAM17 activity, whereas the R177C, as well as the ΔPro, exhibited depleted proteolytic activity (Figure 4J).

Taken together, our results show that the cancer-associated R177C mutation, as well as the lack of the entire pro-domain, leads to impaired ADAM17 maturation and trafficking to the cell surface. By measuring shedding activity by ELISA and proteolytic cleavage on the cell surface as well as in cell lysates, both ADAM17 variants were severely affected in their proteolytic function. Although dimerization seems not to be disturbed in the cancer-associated mutant, this data indicates the fundamental importance of the pro-domain for the proteolytic function of the enzyme and might thereby play a crucial role in cancer development.

## 3. Discussion

The direct involvement of the metalloprotease ADAM17 in inflammation and colon cancer development has been recently shown in several mouse studies [10,13,36]. Thereby the role of ADAM17 in cancer initiation and progression was linked to its shedding ability of specific substrates from the cell surface, which include cytokines, cytokine receptors (e.g., IL-6R), cell adhesion molecules and growth factors important for development and differentiation, like the EGF-R ligands TGFa, Hb-EGF and AREG [9]. Importantly, EGF-R is implicated in the development of many human cancer types [37], in which EGF-R overexpression and increased activity could be assessed [38,39,40]. Since EGF-R activation requires ADAM17 activity [13], it underlines the role of ADAM17 in cancer development and moreover provides a novel therapeutic target. Just recently it could be shown in mouse studies, that ADAM17 is needed for the EGF-R-mediated induction of IL-6 synthesis, which was able to induce tumorigenesis in the colon via IL-6 trans-signaling, a process where also ADAM17 shedding of the IL-6R is required [13].

In this study, we screened human cancer databases to better understand the link between *ADAM17* mutations and cancer progression. Interestingly, we found variants of *ADAM17* listed for various different tumors with the highest percentage of *ADAM17* mutations found in the small and large intestine (1.92% and 1.72%), as well as endometrium (1.69%) and urinary tract (1.58%) (COSMIC database, Sanger Institute). Since recent mouse data pointed to an important role of ADAM17 in colon cancer development [13,36,41], we focused on *ADAM17* mutations found within the large intestine. Although ADAM17 has been a matter of intense study, its regulation of proteolytic activity, as well as substrate recognition in health as well as in disease, is still under debate. Especially within pathological pathways, as discussed here for colon cancer, it is important to fully understand activation but also impairments in ADAM17 function, which might provide us with potential new therapeutic strategies in the future.

In this study, we characterized naturally occurring point mutations found in colon cancer tissue of patients within the pro-, membrane-proximal- and cytoplasmic-domain to unravel regulatory mechanisms of ADAM17 maturation, trafficking and proteolytic activity.

We could evaluate that point mutations within the membrane-proximal-, but also cytoplasmic- domains vary in their proteolytic activity, always showing residual activity towards membrane-bound IL-6R (Figure 2A), pro-TNFa (Figure 2B), as well as a soluble TNFa peptide (Figure 2E). Hence, the D616N mutation within the N-terminal part of the membrane-proximal-domain exhibits a significant impairment in IL-6R shedding (~25% reduction). This underlines the potential importance of this protein domain in recognition of ADAM17 substrates and mediating specificity and proteolytic activity especially towards IL-6R [22]. As indicated in the protein model (Figure 1B), the D616N is pointing to the outside of the protein, which makes the area around the point mutation likely to be involved in mediating IL-6R recognition. The colon-cancer associated point mutation within the cytoplasmic domain (R725H) also exhibits diminished IL-6R shedding (~25% reduction). In general, the R725H mutation seems to get trafficked to the cell membrane in a sufficient manner (Figure 2E; Figure 3C). Overall cell membrane trafficking analyzed by FACS analysis was diminished for all four mutants in comparison to the ADAM17 wt (Figure 3C). However, at least for the two mutations found in the MPD as well as in the cytoplasmic domain, this seems to be sufficient for efficient proteolytic activity. Most strikingly, we found the cancer-associated R177C variant located within the pro-domain of the enzyme severely affected in its proteolytic activity (Figure 2A,B,E) as well as trafficking to the cell membrane (Figure 3C; Figure 4F, middle panel). The ADAM17 pro-domain has been described to be important as an initial inhibitor of the enzyme during translation [42,43], acting as a chaperone during the transport from the ER to the Golgi apparatus [18]. Within the trans-Golgi network, the pro-domain is cleaved by furin but has been shown to be further able to mediate interaction and thus inhibition of ADAM17 until it reaches the cell membrane [19]. This opens novel possibilities to utilize the pro-domain as a specific inhibitor of ADAM17 activity on the cell surface [20,42,43,44]. In the future, this might be useful in the modulation of enhanced EGF-R signaling as found in most cancer types [4,37,44]. In this study, we could evaluate that the R177C variant was similar to a mutant lacking the entire pro-domain (ΔPro) impaired in its activity (Figure 4C–E) as well as in its maturation and cell membrane localization (Figure 4E,F). This led us to the conclusion that this mutation, which has been found in cancer tissue from two separate individuals within the caecum and the brain (COSMIC database, Sanger Institute), severely impairs pro-domain and thus ADAM17 function. This is in line with earlier studies, that have shown defects in secretion of recombinant ADAM17 lacking the pro-domain in insect cells [45]. We here show that the mutation within the pro-domain seems to affect ADAM17 biology in such a great extent, that not only enzyme activity on the cell surface, but also from total lysate was completely abolished (Figure 4J). These data suggest a crucial role of the pro-domain in stability, folding and thus protease activity, further underlining its vital importance for ADAM17 function. The molecular mechanisms of how the pro-domain mediates interaction with the catalytic domain of ADAM17 and is thereby controlling enzyme maturation are unknown. To date, there is no structural data available showing the orientation and interaction of the pro-domain with the catalytic domain. Additionally, within the protein sequence of ADAM17, there are three potential *N*-glycosylation sites available, which suggest that also glycosylation might be an important regulator of ADAM17 activity [35,46]. One of the potential glycosylation sites is in immediate proximity to the mutation (aa177): N-V-S (aa174-aa176). We speculate that this vicinity might have an impact on the glycosylation and thereby further maturation of ADAM17. In a deglycosylation assay (EndoH/PNGaseF) we could show that the R177C variant lacks mature ADAM17 (Figure 4I). Since the pro-domain could still be removed by recombinant furin in an in vitro assay, the mutation does not interfere with cleavage by the proprotein convertase (Figure 4G).

Taken together, all here analyzed cancer-associated variants of ADAM17 show unchanged or diminished proteolytic activity. This seems to be contradictory to the basic understanding of ADAM17-mediated cancer development, where increased EGF-R activity and thus tumorigenesis has been thought to be facilitated by enhanced shedding of EGF-R ligands by ADAM17. At least for the here analyzed ADAM17 variants, their involvement in colon cancer might have different underlying causes. Firstly, it has to be considered that sequencing data found in online databases lack further information on the genetic background of the patients. Hence, it is actually unknown if the here analyzed ADAM17 mutations are actively driving tumorigenesis (‘driver mutation’) or if another mutation, e.g., within the oncogene *KRAS* gene [47] is primarily involved in tumor formation and variants within *ADAM17* are found as a so-called ‘passenger mutations’ [48,49].

Moreover, deficiency of ADAM17 function has strongly been linked to increased inflammation in mouse models with a genetic downregulation of *ADAM17* (hypomorphic ADAM17^ex/ex^) [10]. This augmentation of inflammation within the colon was due to impaired regeneration of the intestinal epithelium in absence of ADAM17 [10]. Paradoxically, increased inflammation has been shown to enhance tumor progression instead of preventing it [50] and thereby is a risk factor for colon cancer [3].

Summarizing, we propose crucial importance of the pro-domain on enzyme maturation and function and suggest ADAM17 as a modulator in colon cancer by its importance in inflammatory events, which have been shown to mediate tumorigenesis. In the future, studies evaluating ADAM17 function in colon cancer development and how interference with ADAM17 function might be beneficial, have to be performed in the respective animal models.

## 4. Material and Methods

### 4.1. Databases

The analyzed cancer-associated ADAM17 variants were found in the databases of IntOGen (Integrative Onco Genomics, Barcelona Biomedical Genomics Lab, Barcelona, Spain), Cosmic (Catalogue of Somatic Mutations in Cancer; Sanger Institute, Cambridge, UK), The Cancer Genome Atlas Program (TCGA; National Institutes of Health, Bethesda, MD, USA) and the International Cancer Genome Consortim (ICGC). These databases list somatically acquired mutations found in tumor tissue of cancer patients.

### 4.2. cDNA Constructs and Cloning

Expression plasmids of murine (m) ADAM17 wildtype (wt), as well as the colon cancer-associated mutants (R177C, D616N, D657A, R725H), were cloned via site-directed mutagenesis PCR utilizing the mADAM17 in the pcDNA3.1 (+) vector as a template. All ADAM17 variants were tagged with a myc encoding sequence on the C-terminus and all constructs were verified by DNA sequencing (GATC Biotech). Plasmids of the ADAM17 substrates murine IL-6R and proTNFα in pcDNA3.1 (+) vector were used for co-transfection together with ADAM17 plasmids for further analyses of ADAM17 enzymatic activity.

### 4.3. Cell Culture

HEK cells deficient for ADAM10 and ADAM17 (A10/A17 dKO) were generated via CRISPR/Cas9 as recently described in [51]. Cells were cultured in Dulbecco’s Modified Eagle’s medium (DMEM) containing 10% heat-inactivated fetal calve serum (FCS) and 1× penicillin/streptomycin (Pen/Strep).

### 4.4. Transfection

In order to ensure reproducibility, the cell count was determined prior to each transfection by Cellometer^®^ Auto T4 Plus (Nexcelom Bioscience, Lawrence, MA, USA). The HEK A10/A17 dKO cells were plated at a density of 3.5 × 10^5^ cells per 6-well. The next day, cells were transfected utilizing polyethylenimine (PEI, 1 µg/µL) in a 1:3 DNA to PEI ratio. After 46 h, the medium was changed and where indicated the cells were stimulated with 200 nM phorbol 12-myristate 13-acetate (PMA) for 2 h and harvested.

### 4.5. Enzyme-Linked Immunosorbent Assay (ELISA)

Cells were transfected (3.5 × 10^5^ cells) as described above with each ADAM17 variant and the respective substrate (IL-6R and pro-TNFα). The cytokines (IL-6R and TNFα) were measured in the cell-free supernatant by ELISA according to the manufacturer’s instructions (mTNFα: ebioscience, Frankfurt am Main, Germany; mIL-6R: R and D Systems, Minneapolis, MN, USA).

### 4.6. Flow Cytometry

HEK cells were transfected as described above. After 24 h, cells were counted and 2.5 × 10^5^ cells were washed three times in ice-cold PBS and blocked for 10 min in PBS containing 1% BSA, incubated with the primary antibodies diluted 1:100 in 1% BSA (in PBS) for 1h at 4 °C. Afterwards, the cell suspension was washed twice with ice-cold PBS containing 1% BSA and incubated with the secondary fluorescence-coupled antibody (diluted 1:100 in PBS containing 1% BSA) for 1 h at 4 °C. Before performing flow cytometry analysis (FACS Canto II, BD Bioscience, Heidelberg, Germany), the cells were washed again twice with ice-cold PBS containing 1% BSA. First, the single cells of the measured 30,000 events were gated using the forward scatter area and forward scatter height. All data analysis was performed using the flow cytometry analysis software FlowJo (Treestar, Ashland, OR, USA). The following antibodies were used: anti-ADAM17 10.1 (1:100; binding ADAM17 extracellular *N*-terminus; polyclonal; generated by Pineda Antikörper-Service, Berlin, Germany), donkey-anti-rabbit Alexa Fluor 488 (1:100; Thermo Fisher Scientific, Waltham, MA, USA; #A21206).

### 4.7. Immunofluorescence Analysis

HEK A10/A17 dKO cells (2.0 × 10^5^ per well) were seeded in a 6-well plate on glass cover slips and transfected as described above. Cells were washed with PBS, fixed with 4% PFA in PBS for 15 min at room temperature and permeabilized with 0.3% Triton X-100 (Sigma-Aldrich, Darmstadt, Germany) in PBS for 30 min. Afterwards, cells were blocked in blocking buffer (2% BSA, 5% heat-inactivated FCS and 0.3% Triton in PBS) for 60 min. The primary antibodies were diluted in blocking buffer to their working concentration (see below) and incubated at 4 °C overnight. Cells were washed three times with PBS containing 0.3% Triton X-100 and incubated with secondary antibodies diluted 1:500 in blocking buffer. Afterwards, cells were washed three times with 0.3% Triton X-100 in PBS, one time with PBS and stained with DAPI as part of the mounting mix consisting of Dabco (Sigma-Aldrich, Darmstadt, Germany) and Mowiol (Merck Millipore, Burlington, MA, USA). Immunofluorescence analyses were performed with a confocal laser scanning microscope (FV1000, Olympus) equipped with a U Plan S Apo 100× oil immersion objective. Digital images were processed using FV10-ASW 4.2 Viewer (Olympus, Tokyo, Japan). Primary antibodies used: anti-PDIA6 (1:750, Abcam, Cambridge, UK; #ab11432), anti-ERp57 (1:100, Abcam, #ab13506), anti-GM130 (1:100, Abcam, #EP892Y), anti-myc (1:250, Cell Signaling, Frankfurt am Main, Germany; clone 9B11 #2276), anti-ADAM17 10.1 (1:100, Pineda Antikörper-Service, Berlin, Germany). Secondary antibodies were purchased from Thermo Fisher Scientific (Waltham, MA, USA): goat-anti-mouse Alexa Fluor 594 (#A11032) and goat-anti-rabbit Alexa Fluor 488 (#A11037).

### 4.8. Western Blot Analysis

HEK cells were transfected as described before and mechanically detached from the culture dish. All cells were washed with ice-cold PBS and lysed in lysis buffer (1% Triton X-100, 150 mM NaCl, 50 mM Tris-HCl pH 7.4), 10 mM 1.10-phenanthroline (Merck, Darmstadt, Germany) and 1× complete protease cocktail inhibitor cocktail (Roche, Basel, Switzerland). The amount of total protein was determined by BCA assay (Thermo Fisher). Aliquots of the lysates were supplemented with 5× SDS sample buffer (0.3 M Tris-HCl, pH 6.8, 10% SDS, 50% glycerol, 5% β-mercaptoethanol, 5% bromophenol blue) and heat inactivated at 95 °C for 5 min. Equal amounts (40µg total protein) were separated by SDS-PAGE and transferred to a PVDF-membrane (Merck Millipore, Burlington, MA, USA).

For the reduced and non-reduced SDS-PAGE, 40 µg of protein was supplemented with 4× NuPAGE LDS Sample Buffer and 10X NuPAGE reducing agent (both Thermo Fisher, #NP0007, #NP0004) and incubated for 10 min at 70 °C. Proteins were separated on a 10% Bis-Tris gel (Thermo Fisher Scientific, Waltham, MA, USA; #NP0301PK2) and blotted as described above.

Following antibodies were used for detection: anti-ADAM17 10.1, anti-ADAM17 18.2 (both Pineda Antikörper-Service), anti-ADAM17 (Abcam, Cambridge, United Kingdom; #ab39162), anti-myc (Cell Signaling, Frankfurt am Main, Germany; clone 9B11 #2276), anti-TNFα (Pineda Antikörper-Service), anti-IL-6R C#1 (Pineda Antikörper-Service, Berlin, Germany), anti-GAPDH (Cell Signaling, Frankfurt am Main, Germany; #2118) and anti-β-actin (Sigma-Aldrich, Darmstadt, Germany; #A5441). As secondary antibodies, goat anti-rabbit HRP and goat anti-mouse HRP (both Dianova, Hamburg, Germany) were used.

### 4.9. Deglycosylation Analysis

HEK dKO cells (2 × 10^6^ cells) were lysed in lysis buffer (see above) for 1 h at 4 °C. Protein amount was determined by BCA assay (Thermo Fisher Scientific, Waltham, MA, USA). Protein (20 µg) was treated with EndoH or PNGaseF (New England Biolabs, Frankfurt am Main, Germany; #P0702S, #P0704S) according to the manufacturer’s instruction.

### 4.10. Immunoprecipitation and Furin Cleavage Assay

Transfected cells were lysed in lysis buffer and protein amount was determined as described above. Afterwards, 0.8 mg protein was incubated with 1.6 µg anti-ADAM17 antibody (Abcam, Cambridge, UK; #ab39162) overnight at 4 °C. In parallel magnetic Protein G Dynabeads (Thermo Fisher Scientific, Waltham, MA, USA; #10004D) were blocked overnight with 1% BSA in PBS. The following day the blocked beads were washed three times in lysis buffer and added to the lysates (1 h, 4 °C). After three washing steps with lysis buffer, furin cleavage assay buffer (100 mM HEPES, 1mM CaCl, 1 mM β-mercaptoethanol, 0.5% Triton X-100) with 1 U furin (New England Biolabs, Frankfurt am Main, Germany; #P8077S) was added and incubated for 30 min at 30 °C. 5× SDS sample buffer was added and boiled. The protein was further analyzed via Western blot.

### 4.11. Live Cell Surface ADAM17 Activity Assay

HEK dKO cells (2 × 10^6^ cells) were seeded onto a 10 cm culture dish and transfected on the next day (see protocol above). After 24 h the cells were trypsinized and seeded onto a 96 well plate (2 × 10^5^ cells per 96 well). The following day, the media was removed and PBS was added containing 20 µM of the quenched fluorogenic peptide (Abz-LAQAVRSSSR-Dpa; ADAM17 (TACE) substrate IV (Calbiochem, Merck, Darmstadt, Germany #616407)). Cell surface activity of ADAM17 was measured every 30 s over 120 min at emission 405 nm and extinction 320 nm with a Tecan plate reader (Tecan Infinite 200 Pro) and determined using the area under the curve (AUC) of the fluorescence signal over time.

### 4.12. Cell Lysate ADAM17 Activity Assay

HEK dKO cells (2 × 10^6^ cells) were seeded and transfected as described above. After 48 h, the cells were mechanically detached and lysed in lysis buffer (1% Triton X-100, 150 mM NaCl, 50 mM Tris-HCl pH7.4), supplemented with 4% glycerol, 10 mM 1.10-phenanthroline and 1× complete protease inhibitor cocktail EDTA-free (Roche, Basel, Switzerland) for 1 h at 4 °C. The protein concentration was determined using the BCA assay (Thermo Fisher Scientific, Waltham, MA, USA). Aliquots of 150 µg protein were transferred to a black 96 well plate (Thermo Fisher Scientific, Waltham, MA, USA) and 20 µM quenched fluorogenic peptide (Abz-LAQAVRSSSR-Dpa; ADAM17 (TACE) substrate IV (Calbiochem, Merck, Darmstadt, Germany; #616407)) was added and incubated for 30 min at 37 °C. ADAM17 activity was measured at emission 405 nm and extinction 320 nm with a Synergy HT microplate reader (BioTec, Winooski, VT, USA).

### 4.13. Structural Analysis

ADAM17 was modelled using Swiss-Model Expasy. As a template for ADAM17, the available structure of ADAM10 was exploited [52]. The pro-domain was placed to cover the active site. The missing C-terminal elongation (dotted line Figure 1B), the transmembrane helix and the cytoplasmic domain were also added in cartoon style. The dissection into the different domains (protease, disintegrin, cysteine-rich) was performed according to Uniprot annotation (ID: P78536). Imaging was performed using UCSF Chimera [53].

### 4.14. Data Analysis and Statistics

All values are expressed as the mean ± SEM. For data analysis Excel (Microsoft, Seattle, WA, USA) and GraphPad Prism version 7 (GraphPad Software for Mac, San Diego, CA, USA) were used. Differences among mean values were analyzed by two-tailed, unpaired Student t-test or one-way ANOVA, followed by a Dunnett’s multiple comparison test where applicable. In all analyses, the null hypothesis was rejected at *p* < 0.05 with * < 0.05, ** < 0.01, *** < 0.005, **** < 0.001.

## Abbreviations

aa	amino acid
ADAM17	A Disintegrin and Metalloproteinase 17
CD	cytoplasmic domain
COSMIC	Catalogue of Somatic Mutations in Cancer
dKO	double knock-out
ds	downstream
EGF-R	epidermal growth factor receptor
ELISA	Enzyme-Linked Immunosorbent Assay
ER	endoplasmic reticulum
FGF-2	fibroblast growth factor-2
FSC	forward scatter
HEK	human embryonic kidney cells
ICGC	International Cancer Genome Consortium
IL-6R	interleukin-6 receptor
IntOGen	Integrative Onco Genomics
kDa	kilo Dalton
MPD	membrane-proximal domain
PD	pro-domain
PDI	protein disulfide isomerase
PMA	phorbol 12-myristate 13-acetate
sTNFα	soluble tumor necrosis factor alpha
TCGA	The Cancer Genome Atlas Program
us	upstream
VEGF	vascular endothelial growth factor
wt	wildtype

## References

[1] Siegel R., Desantis C., Jemal A. (2014). Colorectal cancer statistics, 2014. CA Cancer J. Clin..

[2] Rustgi A.K. (2007). The genetics of hereditary colon cancer. Genes Dev..

[3] Terzic J., Grivennikov S., Karin E., Karin M. (2010). Inflammation and colon cancer. Gastroenterology.

[4] Sibilia M., Kroismayr R., Lichtenberger B.M., Natarajan A., Hecking M., Holcmann M. (2007). The epidermal growth factor receptor: From development to tumorigenesis. Differ. Res. Biol. Divers..

[5] Egger B., Buchler M.W., Lakshmanan J., Moore P., Eysselein V.E. (2000). Mice harboring a defective epidermal growth factor receptor (waved-2) have an increased susceptibility to acute dextran sulfate-induced colitis. Scand. J. Gastroenterol..

[6] Black R.A., Rauch C.T., Kozlosky C.J., Peschon J.J., Slack J.L., Wolfson M.F., Castner B.J., Stocking K.L., Reddy P., Srinivasan S. (1997). A metalloproteinase disintegrin that releases tumour-necrosis factor-alpha from cells. Nature.

[7] Moss M.L., Jin S.L., Milla M.E., Bickett D.M., Burkhart W., Carter H.L., Chen W.J., Clay W.C., Didsbury J.R., Hassler D. (1997). Cloning of a disintegrin metalloproteinase that processes precursor tumour-necrosis factor-alpha. Nature.

[8] Blobel C.P. (2005). ADAMs: Key components in EGFR signalling and development. Nat. Rev. Mol. Cell Biol..

[9] Zunke F., Rose-John S. (2017). The shedding protease ADAM17: Physiology and pathophysiology. Biochim. Biophys. Acta.

[10] Chalaris A., Adam N., Sina C., Rosenstiel P., Lehmann-Koch J., Schirmacher P., Hartmann D., Cichy J., Gavrilova O., Schreiber S. (2010). Critical role of the disintegrin metalloprotease ADAM17 for intestinal inflammation and regeneration in mice. J. Exp. Med..

[11] Blaydon D.C., Biancheri P., Di W.L., Plagnol V., Cabral R.M., Brooke M.A., van Heel D.A., Ruschendorf F., Toynbee M., Walne A. (2011). Inflammatory skin and bowel disease linked to ADAM17 deletion. N. Engl. J. Med..

[12] Peschon J.J., Slack J.L., Reddy P., Stocking K.L., Sunnarborg S.W., Lee D.C., Russell W.E., Castner B.J., Johnson R.S., Fitzner J.N. (1998). An essential role for ectodomain shedding in mammalian development. Science.

[13] Schmidt S., Schumacher N., Schwarz J., Tangermann S., Kenner L., Schlederer M., Sibilia M., Linder M., Altendorf-Hofmann A., Knösel T. (2018). ADAM17 is required for EGF-R–induced intestinal tumors via IL-6 trans-signaling. J. Exp. Med..

[14] Jostock T., Mullberg J., Ozbek S., Atreya R., Blinn G., Voltz N., Fischer M., Neurath M.F., Rose-John S. (2001). Soluble gp130 is the natural inhibitor of soluble interleukin-6 receptor transsignaling responses. Eur. J. Biochem..

[15] Pagano E., Borrelli F., Orlando P., Romano B., Monti M., Morbidelli L., Aviello G., Imperatore R., Capasso R., Piscitelli F. (2017). Pharmacological inhibition of MAGL attenuates experimental colon carcinogenesis. Pharmacol. Res..

[16] Yu W., Ma Y., Shankar S., Srivastava R.K. (2017). SATB2/beta-catenin/TCF-LEF pathway induces cellular transformation by generating cancer stem cells in colorectal cancer. Sci. Rep..

[17] Wei L.H., Chou C.H., Chen M.W., Rose-John S., Kuo M.L., Chen S.U., Yang Y.S. (2013). The role of IL-6 trans-signaling in vascular leakage: Implications for ovarian hyperstimulation syndrome in a murine model. J. Clin. Endocrinol. Metab..

[18] Leonard J.D., Lin F., Milla M.E. (2005). Chaperone-like properties of the prodomain of TNFalpha-converting enzyme (TACE) and the functional role of its cysteine switch. Biochem. J..

[19] Wong E., Maretzky T., Peleg Y., Blobel C.P., Sagi I. (2015). The Functional Maturation of A Disintegrin and Metalloproteinase (ADAM) 9, 10, and 17 Requires Processing at a Newly Identified Proprotein Convertase (PC) Cleavage Site. J. Biol. Chem..

[20] Wong E., Cohen T., Romi E., Levin M., Peleg Y., Arad U., Yaron A., Milla M.E., Sagi I. (2016). Harnessing the natural inhibitory domain to control TNFalpha Converting Enzyme (TACE) activity in vivo. Sci. Rep..

[21] Moss M.L., Minond D. (2017). Recent Advances in ADAM17 Research: A Promising Target for Cancer and Inflammation. Mediat. Inflamm..

[22] Lorenzen I., Lokau J., Dusterhoft S., Trad A., Garbers C., Scheller J., Rose-John S., Grotzinger J. (2012). The membrane-proximal domain of A Disintegrin and Metalloprotease 17 (ADAM17) is responsible for recognition of the interleukin-6 receptor and interleukin-1 receptor II. FEBS Lett..

[23] Reddy P., Slack J.L., Davis R., Cerretti D.P., Kozlosky C.J., Blanton R.A., Shows D., Peschon J.J., Black R.A. (2000). Functional analysis of the domain structure of tumor necrosis factor-alpha converting enzyme. J. Biol. Chem..

[24] Doedens J.R., Mahimkar R.M., Black R.A. (2003). TACE/ADAM-17 enzymatic activity is increased in response to cellular stimulation. Biochem. Biophys. Res. Commun..

[25] Le Gall S.M., Maretzky T., Issuree P.D., Niu X.D., Reiss K., Saftig P., Khokha R., Lundell D., Blobel C.P. (2010). ADAM17 is regulated by a rapid and reversible mechanism that controls access to its catalytic site. J. Cell Sci..

[26] Maretzky T., Evers A., Zhou W., Swendeman S.L., Wong P.M., Rafii S., Reiss K., Blobel C.P. (2011). Migration of growth factor-stimulated epithelial and endothelial cells depends on EGFR transactivation by ADAM17. Nat. Commun..

[27] Schwarz J., Broder C., Helmstetter A., Schmidt S., Yan I., Muller M., Schmidt-Arras D., Becker-Pauly C., Koch-Nolte F., Mittrucker H.W. (2013). Short-term TNFalpha shedding is independent of cytoplasmic phosphorylation or furin cleavage of ADAM17. Biochim. Biophys. Acta.

[28] Cabron A.S., El Azzouzi K., Boss M., Arnold P., Schwarz J., Rosas M., Dobert J.P., Pavlenko E., Schumacher N., Renne T. (2018). Structural and Functional Analyses of the Shedding Protease ADAM17 in HoxB8-Immortalized Macrophages and Dendritic-like Cells. J. Immunol..

[29] Mullberg J., Schooltink H., Stoyan T., Gunther M., Graeve L., Buse G., Mackiewicz A., Heinrich P.C., Rose-John S. (1993). The soluble interleukin-6 receptor is generated by shedding. Eur. J. Immunol..

[30] Soond S.M., Everson B., Riches D.W., Murphy G. (2005). ERK-mediated phosphorylation of Thr735 in TNFalpha-converting enzyme and its potential role in TACE protein trafficking. J. Cell Sci..

[31] Killock D.J., Ivetic A. (2010). The cytoplasmic domains of TNFalpha-converting enzyme (TACE/ADAM17) and L-selectin are regulated differently by p38 MAPK and PKC to promote ectodomain shedding. Biochem. J..

[32] Xu P., Derynck R. (2010). Direct activation of TACE-mediated ectodomain shedding by p38 MAP kinase regulates EGF receptor-dependent cell proliferation. Mol. Cell.

[33] Xu P., Liu J., Sakaki-Yumoto M., Derynck R. (2012). TACE activation by MAPK-mediated regulation of cell surface dimerization and TIMP3 association. Sci. Signal..

[34] Fan H., Turck C.W., Derynck R. (2003). Characterization of growth factor-induced serine phosphorylation of tumor necrosis factor-alpha converting enzyme and of an alternatively translated polypeptide. J. Biol. Chem..

[35] Peiretti F., Canault M., Deprez-Beauclair P., Berthet V., Bonardo B., Juhan-Vague I., Nalbone G. (2003). Intracellular maturation and transport of tumor necrosis factor alpha converting enzyme. Exp. Cell Res..

[36] Mustafi R., Dougherty U., Mustafi D., Ayaloglu-Butun F., Fletcher M., Adhikari S., Sadiq F., Meckel K., Haider H.I., Khalil A. (2017). ADAM17 is a Tumor Promoter and Therapeutic Target in Western Diet-associated Colon Cancer. Clin. Cancer Res..

[37] Hynes N.E., Lane H.A. (2005). ERBB receptors and cancer: The complexity of targeted inhibitors. Nat. Rev. Cancer.

[38] Spano J.P., Lagorce C., Atlan D., Milano G., Domont J., Benamouzig R., Attar A., Benichou J., Martin A., Morere J.F. (2005). Impact of EGFR expression on colorectal cancer patient prognosis and survival. Ann. Oncol. Off. J. Eur. Soc. Med. Oncol..

[39] Haraldsdottir S., Bekaii-Saab T. (2013). Integrating anti-EGFR therapies in metastatic colorectal cancer. J. Gastrointest. Oncol..

[40] Ohgaki H., Dessen P., Jourde B., Horstmann S., Nishikawa T., Di Patre P.L., Burkhard C., Schuler D., Probst-Hensch N.M., Maiorka P.C. (2004). Genetic pathways to glioblastoma: A population-based study. Cancer Res..

[41] Srivatsa S., Paul M.C., Cardone C., Holcmann M., Amberg N., Pathria P., Diamanti M.A., Linder M., Timelthaler G., Dienes H.P. (2017). EGFR in Tumor-Associated Myeloid Cells Promotes Development of Colorectal Cancer in Mice and Associates With Outcomes of Patients. Gastroenterology.

[42] Gonzales P.E., Solomon A., Miller A.B., Leesnitzer M.A., Sagi I., Milla M.E. (2004). Inhibition of the tumor necrosis factor-alpha-converting enzyme by its pro domain. J. Biol. Chem..

[43] Li X., Yan Y., Huang W., Yang Y., Wang H., Chang L. (2009). The regulation of TACE catalytic function by its prodomain. Mol. Biol. Rep..

[44] Saad M.I., Alhayyani S., McLeod L., Yu L., Alanazi M., Deswaerte V., Tang K., Jarde T., Smith J.A., Prodanovic Z. (2019). ADAM17 selectively activates the IL-6 trans-signaling/ERK MAPK axis in KRAS-addicted lung cancer. EMBO Mol. Med..

[45] Milla M.E., Leesnitzer M.A., Moss M.L., Clay W.C., Carter H.L., Miller A.B., Su J.L., Lambert M.H., Willard D.H., Sheeley D.M. (1999). Specific sequence elements are required for the expression of functional tumor necrosis factor-alpha-converting enzyme (TACE). J. Biol. Chem..

[46] Chavaroche A., Cudic M., Giulianotti M., Houghten R.A., Fields G.B., Minond D. (2014). Glycosylation of a disintegrin and metalloprotease 17 affects its activity and inhibition. Anal. Biochem..

[47] Kinzler K.W., Vogelstein B. (1996). Lessons from hereditary colorectal cancer. Cell.

[48] McFarland C.D., Yaglom J.A., Wojtkowiak J.W., Scott J.G., Morse D.L., Sherman M.Y., Mirny L.A. (2017). The Damaging Effect of Passenger Mutations on Cancer Progression. Cancer Res..

[49] Pon J.R., Marra M.A. (2015). Driver and passenger mutations in cancer. Annu. Rev. Pathol..

[50] Dvorak H.F. (1986). Tumors: Wounds that do not heal. Similarities between tumor stroma generation and wound healing. N. Engl. J. Med..

[51] Riethmueller S., Ehlers J.C., Lokau J., Dusterhoft S., Knittler K., Dombrowsky G., Grotzinger J., Rabe B., Rose-John S., Garbers C. (2016). Cleavage Site Localization Differentially Controls Interleukin-6 Receptor Proteolysis by ADAM10 and ADAM17. Sci. Rep..

[52] Seegar T.C.M., Killingsworth L.B., Saha N., Meyer P.A., Patra D., Zimmerman B., Janes P.W., Rubinstein E., Nikolov D.B., Skiniotis G. (2017). Structural Basis for Regulated Proteolysis by the alpha-Secretase ADAM10. Cell.

[53] Pettersen E.F., Goddard T.D., Huang C.C., Couch G.S., Greenblatt D.M., Meng E.C., Ferrin T.E. (2004). UCSF Chimera—A visualization system for exploratory research and analysis. J. Comput. Chem..

